# Evolution of Metapostnotum in Flat Wasps (Hymenoptera, Bethylidae): Implications for Homology Assessments in Chrysidoidea

**DOI:** 10.1371/journal.pone.0140051

**Published:** 2015-10-14

**Authors:** Ricardo Kawada, Geane O. Lanes, Celso O. Azevedo

**Affiliations:** Departamento de Ciências Biológicas, Universidade Federal do Espírito Santo, Vitória, ES, Brazil; Trier University, GERMANY

## Abstract

Some authors in the past based their conclusions about the limits of the metapostnotum of Chrysidoidea based on the position of the mesophragmo-metaphragmal muscle, rather than aspects of the skeleton and musculature associated with the metapectal-propodeal complex. The latter character system suggests another interpretation of the metapostnotum delimitation. Given this scenario, the main goal of this work is to present a new perspective on the metapostnotum in Chrysidoidea, especially Bethylidae, helping to resolve questions related to the evolution of the metapostnotum. This is based on homologies established by associating of insertion points of ph2-ph3 and ph3-T2 muscles with the delimitation of the respective sclerite the muscles insert into. Our results indicate that, according the position of the metaphragmal muscles, the metapostnotum in Bethylidae is medially expanded in the propodeal disc and has different forms of configuration. Internally, the limits of the metapostnotum can be tracked by the shape of the mesopostnotum, and vice versa. Thus, the anteromedian area of the propodeal disc sensu Evans was reinterpreted in the current study as the metapostnotum. In conjunction with associated structures, we provide evidence to clarify the relationships between the families within Chrysidoidea, although certain families like Embolemidae, Dryinidae and Chrysididae exhibit extreme modifications of the condition found in Aculeata, as observed in Bethylidae. We review the terminology used to describe anatomical features on the metapectal-propodeal complex in Bethylidae in general, and provide a list of recommended terms in accordance with the online Hymenoptera Anatomy Ontology. The morphology of the studied subfamilies are illustrated. Studies that focus on a single structure, across a larger number of taxa, are more insightful and present specific questions that can contribute to broader issues, thus providing a better understanding of the morphology and evolution of insects.

## Introduction

The dorsal muscles of pterothoracic segments of winged insects are usually well developed. The ends of these muscles are attached to internal projections of the cuticle called phragmas. Following Snodgrass [1], the phragmas are intersegmental inflections formed from the **antecosta** (**ac**) of the **mesonotum** (**n2**), **metanotum** (**n3**) and **first abdominal tergum** (**T1**). The antecosta is an internal elevation near the anterior margin of each notum, which is externally represented by the **antecostal sulcus** (**acs**). The narrow rim located anteriorly to the antecostal sulcus is known as **acrotergite** (**atg**), and its fusion with the antecosta of the mesothorax and metathorax is known as the **mesopostnotum** (**pn2**) and **metapostnotum** (**pn3**) [2], respectively.

According to Snodgrass [3], the ground-plan condition of the metapostnotum in Hymenoptera is a transverse medially continuous tergite that is articulated or laterally fused with the **metepimeron** (**epm3**) and anterior to the first abdominal spiracles (**propodeal spiracle** in Apocrita, **prs**). The metapostnotum is anterior to the metanotum and closely associated with the first abdominal tergum posteriorly (**propodeum** in Apocrita, **pr**), which is separated by the **metapostnotal-propodeal suture** (**mps**). The **metaphragma** (**ph3**) arises from the antecosta of the first abdominal tergum in the posterior margin of the metapostnotum and attaches two pair of dorsal longitudinal muscles, between the meso- and metapostnotum and between the metapostnotum and **second abdominal tergum** (**T2**).

The combination of characters of the metapostnotum and associated structures based in a cladistics framework can help characterize the vast majority of previously established lineages of “Symphyta." Whitfield et al., [4] reported that Siricoidea, Cephoidea and Xiphydrioidea share a medially divided metapostnotum; Orussoidea have a continuous metapostnotum fully fused with T1; Xyeloidea have a continuous metapostnotum partially fused with T1; and Tenthredinoidea have a medially continuous metapostnotum and with different fusion conditions with T1. Thus, in the basal groups of Hymenoptera the metaphragma can vary from absent to well developed.

In Apocrita, the structure + function of the metapostnotum changed because of the partial fusion between the **metathorax** (**th3**) and first abdominal tergum, which forms the propodeum, and hinders the visualization of metapostnotum limits. Whitfield et al. [4] suggested the non-aculeate Apocrita exhibit a different evolutionary pattern in the metapostnotum from basal groups of Hymenoptera. In the non-aculeate Apocrita groups, the metapostnotum is never truly divided medially, although it is often depressed medially and reduced to a narrow strip anterior to the metapostnotal-propodeal suture. In addition, the metapostnotum may be hidden in dorsal view by the overhanging metanotum or totally absent (especially in Chalcidoidea). Alternatively, it may occur as it does in many Apocrita, in which the metapostnotum can be difficult to identify externally because of a loss of definition in the suture between the metapostnotum and propodeum [4].

Brothers [5] suggested the Apoidea are the only group that exhibits a posteriorly expanded metapostnotum, since the posterior insertion of the **mesophragmo-metaphragmal muscle** (**ph2-ph3**) is over half the length of the **mesophragma** (**ph2**). Based on Bethylidae, Brothers [5] concluded that the metapostnotum in Chrysidoidea could be visualized externally only by its lateral remnants due to the anterior insertion of ph2-ph3 in the mesophragma and because the metaphragma is reduced or absent.

Whitfield et al. [4] and Brothers [5] suggested another evolutionary scenario for the metapostnotum based on their conclusions on the position of the mesophragmo-metaphragmal muscle. However, they did not consider relevant aspects of the skeleton and musculature associated with the metapectal-propodeal complex in Apocrita. Although Vilhelmsen [6] and Vilhelmsen et al. [7] have not made inferences on metapostnotum evolution, they analyzed the other muscle associated with the metaphragma, the **metaphragmo-second abdominal tergal muscle** (**ph3-T2**), which can also be used in the delimitation of the metapostnotum.

Although Matsuda [8] argued that the musculature and exoskeleton developed independently, and homologous muscles could be attached to different parts of the insects’ exoskeletons, several authors (e.g., [7, 9, 10, 11]) have defined homologies for Hymenoptera external structures based on musculature. According to Gibson [10], although comparative morphology requires a phylogenetic framework to be testable, most of the external resources of the insect’s exoskeleton reflect its internal musculature; therefore, knowledge of the musculature may indicate homology among externally different structures as well as discrepancies in the observation or interpretation of the external structure. Thus, we use here the musculature associated with the mesopostnotum of Bethylidae as anatomical evidence to present a new perspective, and attempt to resolve questions related to the evolution of the metapostnotum in Chrysidoidea.

## Materials and Methods

### Sampling

The classification of Alencar and Azevedo [12] was used to represent the subfamilies of Bethylidae, which were used with other Hymenoptera families in this study. Specimens (S1 Table) studied are from Universidade Federal do Espírito Santo, Vitória, Brazil (UFES), California Academy of Sciences, California, USA, (CASC), and Queen Sirikit Botanic Garden, Chaing Mai, Thailand (QSBG). Ethanol-stored, critical point-dried, and air-dried specimens were used for morphological observations.

### Dissection techniques

The methods of Gibson [9] and Vilhelmsen et al. [7] were used in the study of the skeleton and musculature. The specimens were immersed in 100% alcohol one day prior to dissection for complete dehydration and then critical-point dried for maximal preservation of the internal parts in their natural state. After the drying process, the specimens were transferred to an adhesive putty (Blu Tack) [13], which allowed better handling during dissection. All the dissections were performed using pieces cut out with a razor blade and 000 entomological pins. The specimens were dissected so that the metapectal-propodeal complex + metasoma were preserved for the study. The remaining parts, such as the head, legs and wings, were glued on a card along with information on the sample’s origin and stored as vouchers. All specimens were dissected as closely as possible to the mid-sagittal region of the metapleural-propodeal complex + petiole for observation and later illustration of the structures.

### Morphological character circumscription

Character circumscription was conducted following well-established lines of evidence on morphological data, such as topological correspondence between observed structures [14]. The characters were treated as hypotheses of primary homology following De Pinna [15]. The anatomical data are consistent with the Hymenoptera Anatomy Ontology project [16, 17], determined using the proofing tool available through the Hymenoptera Glossary (HAO) and the literature that addresses the specific morphology of each Hymenoptera family. Abbreviations of anatomical structures used in figures are listed in S2 Table with a brief historical review of the structure terminology used.

### Image acquisition

The specimens were photographed under a Leica Z16 APO stereomicroscope coupled to a Leica DFC 2 video camera by Leica Microsystems (Switzerland). The equipment used for data storage was a high-performance notebook with Windows 7 Professional operating system and an Intel (R) Xeon (R) central processing unit (CPU), and two different software programs were used to combine the images: Leica Application Suite V3.6.0 by Leica Microsystems (Switzerland), which uses the parameters max. process, precision optimize, and 15–40 patch size to combine images, and Helicon Focus (HeliconSoft), which uses the parameters A, B or C method; 100% full resolution; 1–4 radius; 1 smoothing; and 600 DPI. The illumination of all specimens was performed following Buffington et al. [18], Keer et al. [19] and Buffington and Gates [20]. For more efficient light diffusion, a dome was used along with a tracing paper ring, which was placed around specimens. The final images were edited using adjustments (e.g., levels and shadows/highlights), tools (healing brush, clone stamp, etc.) and filters (unsharp mask). The highlights used for the characters, including arrows and different vector colors, were edited in Adobe Illustrator®.

## Results

**Fig 1:**
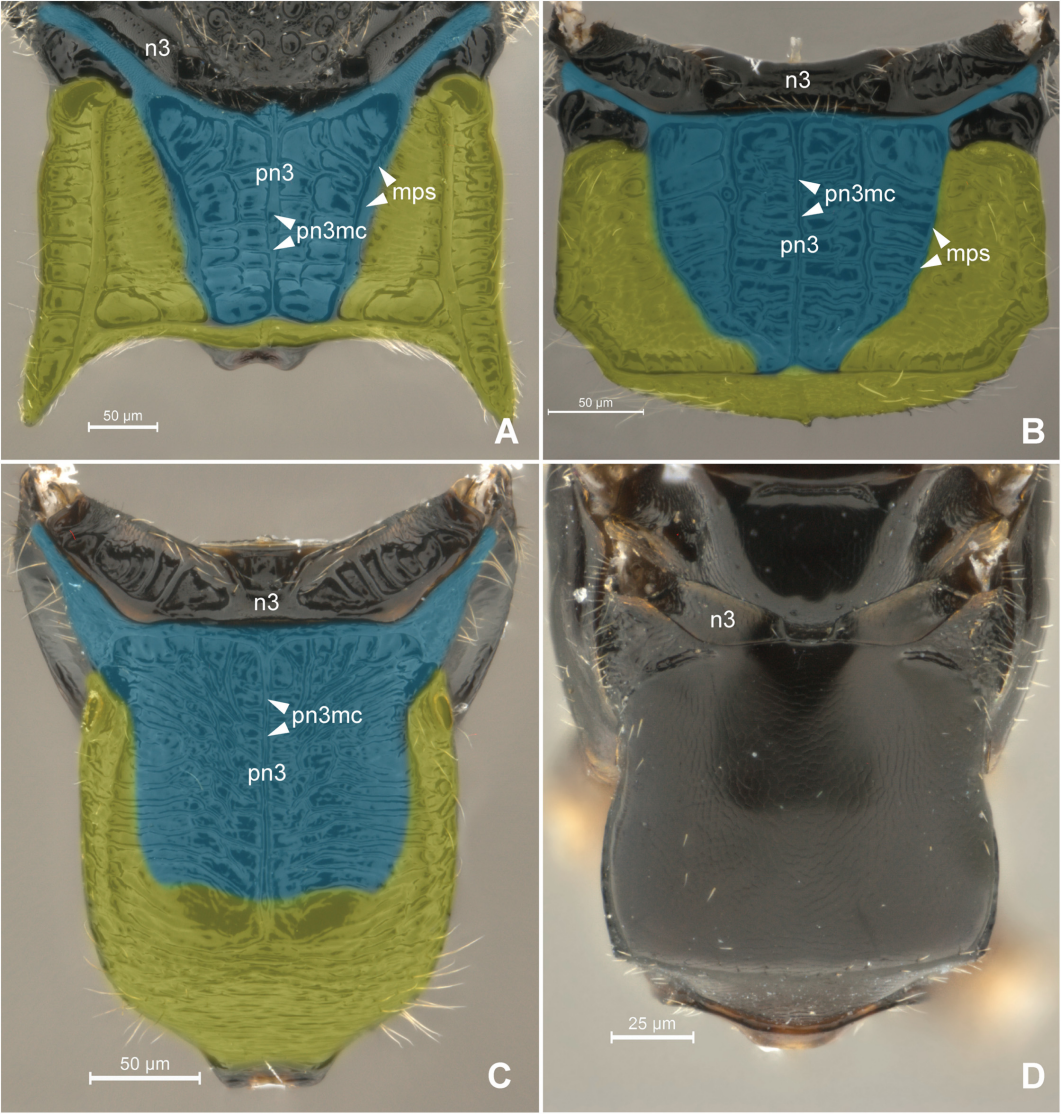
Detail of Metapectal-propodeal complex. 1A *Heterocoelia* sp. (Mesitiinae), 1B *Chlorepyris* sp. (Epyrinae), 1C *Pseudisobrachium* sp. (Pristocerinae), 1D *Sclerodermus* sp. (Scleroderminae). (A-D) Metapectal-propodeal complex, dorsal view. Blue = metapostnotum; yellow = first abdominal tergo. Scale bar in the figure.

**Fig 2:**
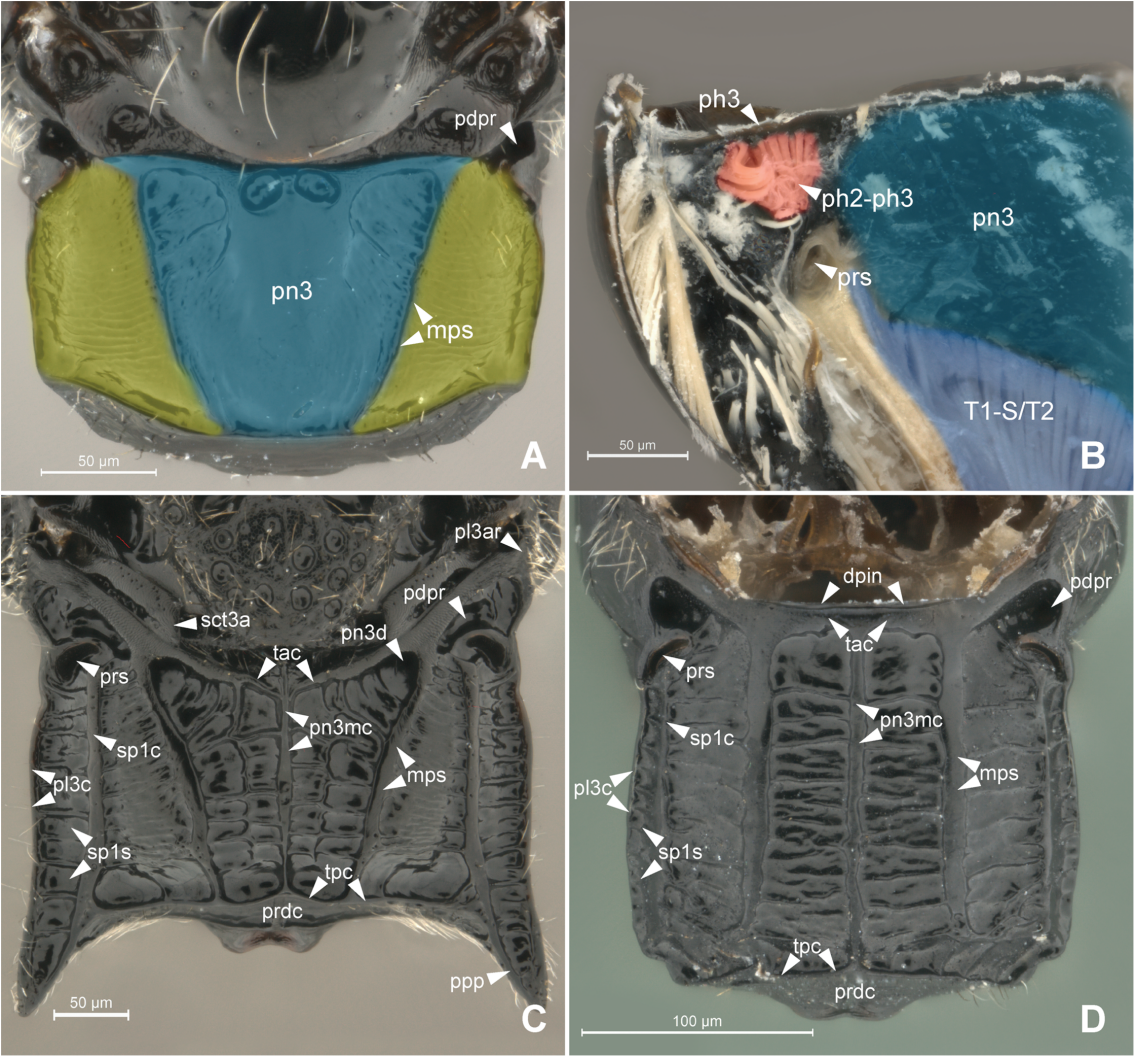
Detail of Metapectal-propodeal complex. 2A *Prosierola* sp. (Bethylinae), 2B *Pristocera* sp. (Pristocerinae), 2C *Heterocoelia* sp. (Mesitiinae), 2D *Epyris* sp. (Epyrinae). (A) Metapectal-propodeal complex, dosal view. Blue = metapostnotum; yellow = first abdominal tergo. (B) Metapectal-propodeal complex, internal anteroposterior view. Blue (upper) = metapostnotum; blue (lower) = propodeo-second abdominal segment muscle; red = mesophragmo-metaphragmal muscle. (C-D) Metapectal-propodeal complex, dorsal view. Scale bar in the figure.

**Fig 3:**
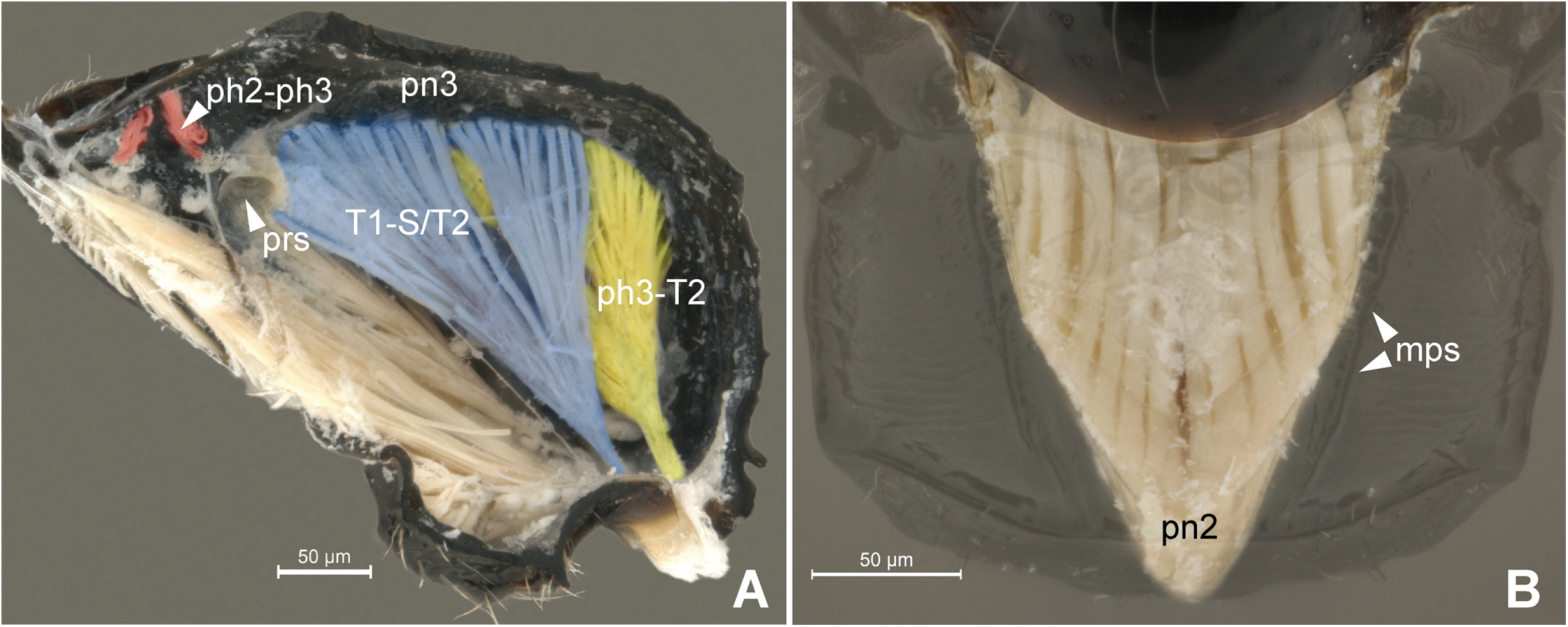
Musculature of Metapectal-propodeal complex. 3A *Pristocera* sp. (Pristocerinae), 3B *Prosierola* sp. (Bethylinae). (A) Metapectal-propodeal complex, internal medial view. Red = mesophragmo-metaphragmal muscle. (B) Emphasis on the prophragmo-mesophragmal muscle through of metapectal-propodeal complex, using low opacity with image editing tool, dorsal view. Scale bar in the figure.

According to the position of the metaphragmal muscles, we observed that the metapostnotum (**pn3**; Figs 1A–1C, 2A, 2B and 3A) in Bethylidae is medially expanded in the propodeal disc, resulting a variety of morphologically distinct forms (Figs 1 and 2A).

Anteriorly, the metapostnotum is limited by the mesophragmo-metaphragmal muscle (**ph2-ph3**; Figs 2B and 3A) that is inserted posterior to the **metascutellar arm** (**sct3a**; Fig 2C), and anterior to the **prespiracular depression of the propodeum** (**pdpr**; Fig 2C and 2D); laterally by the **dorsal propodeal inflection** (**dpin**; Fig 2D). Laterally, the metaposnotum is limited by the **propodeo-second abdominal segment muscle** (**T1-S/T2**; Figs 3A and 2B) that arises posterior to the propodeal spiracle along the metapostnotal-propodeal suture. Posteriorly, the metapostnotum is limited by the metaphragmo-second abdominal tergal muscle (**ph3-T2**; Fig 3A) that arises anterior to the **transverse posterior carina of the propodeum** (**tpc**; Fig 2C and 2D) near the **propodeal declivity** (**prdc**; Fig 2C and 2D).

**Fig 4:**
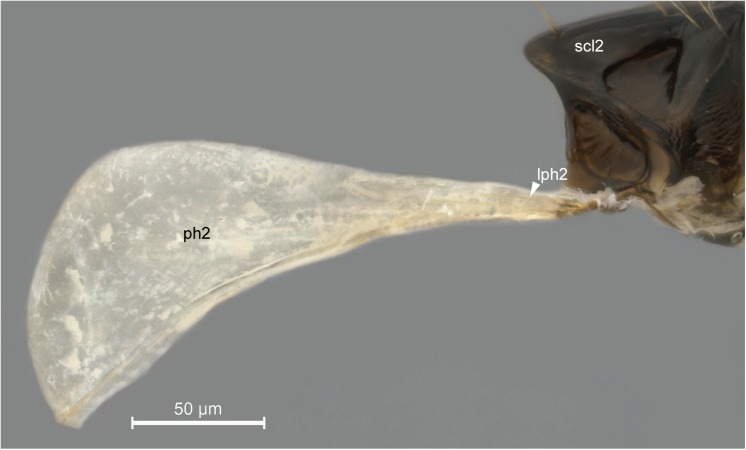
Detail of mesopostnotum (mesolaterophragma and mesophragma), lateral view. *Pristocera* sp. (Pristocerinae). Scale bar in the figure.

**Fig 5:**
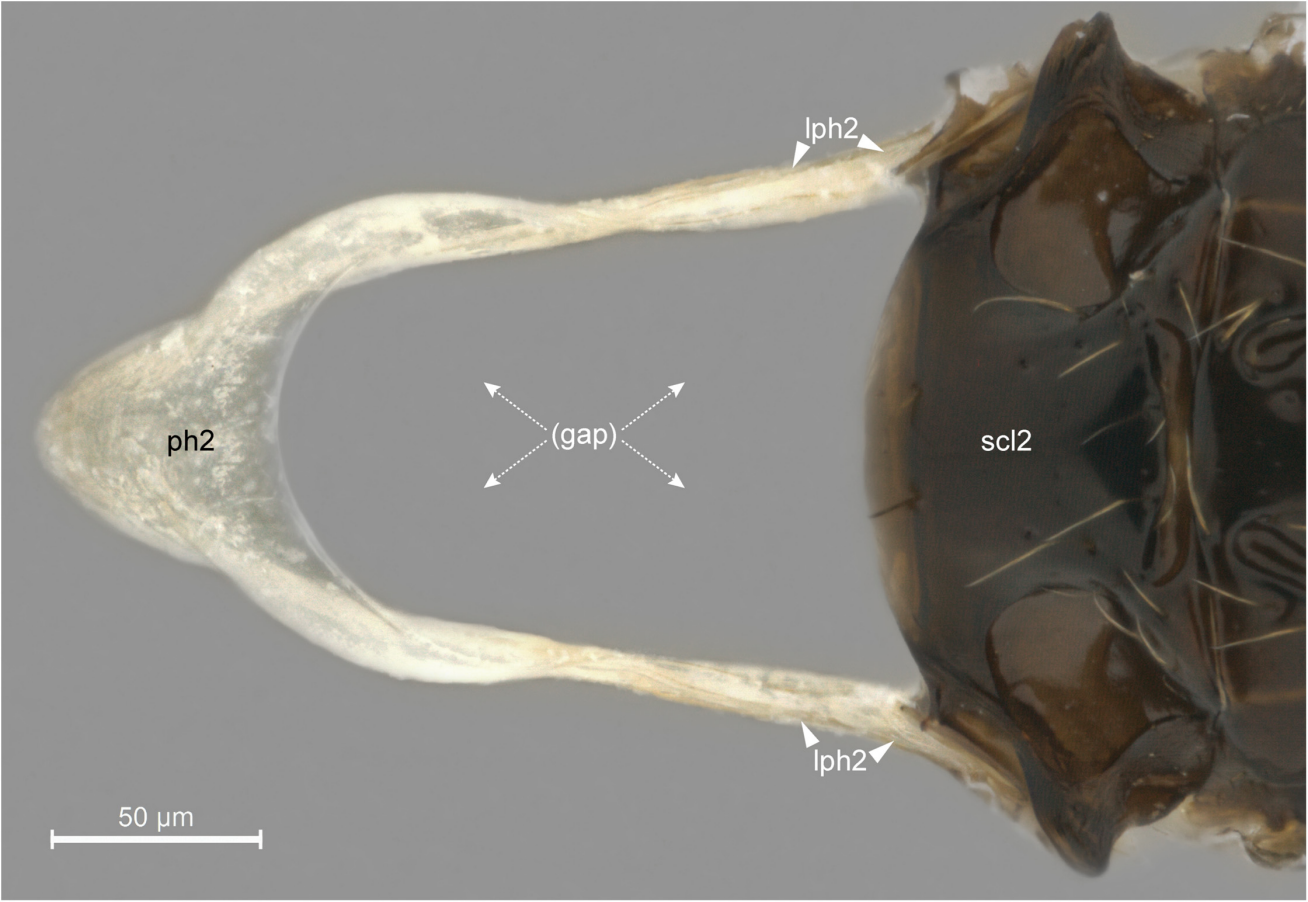
Detail of mesopostnotum, dorsal view. *Pristocera* sp. (Pristocerinae). Scale bar in the figure.

**Fig 6:**
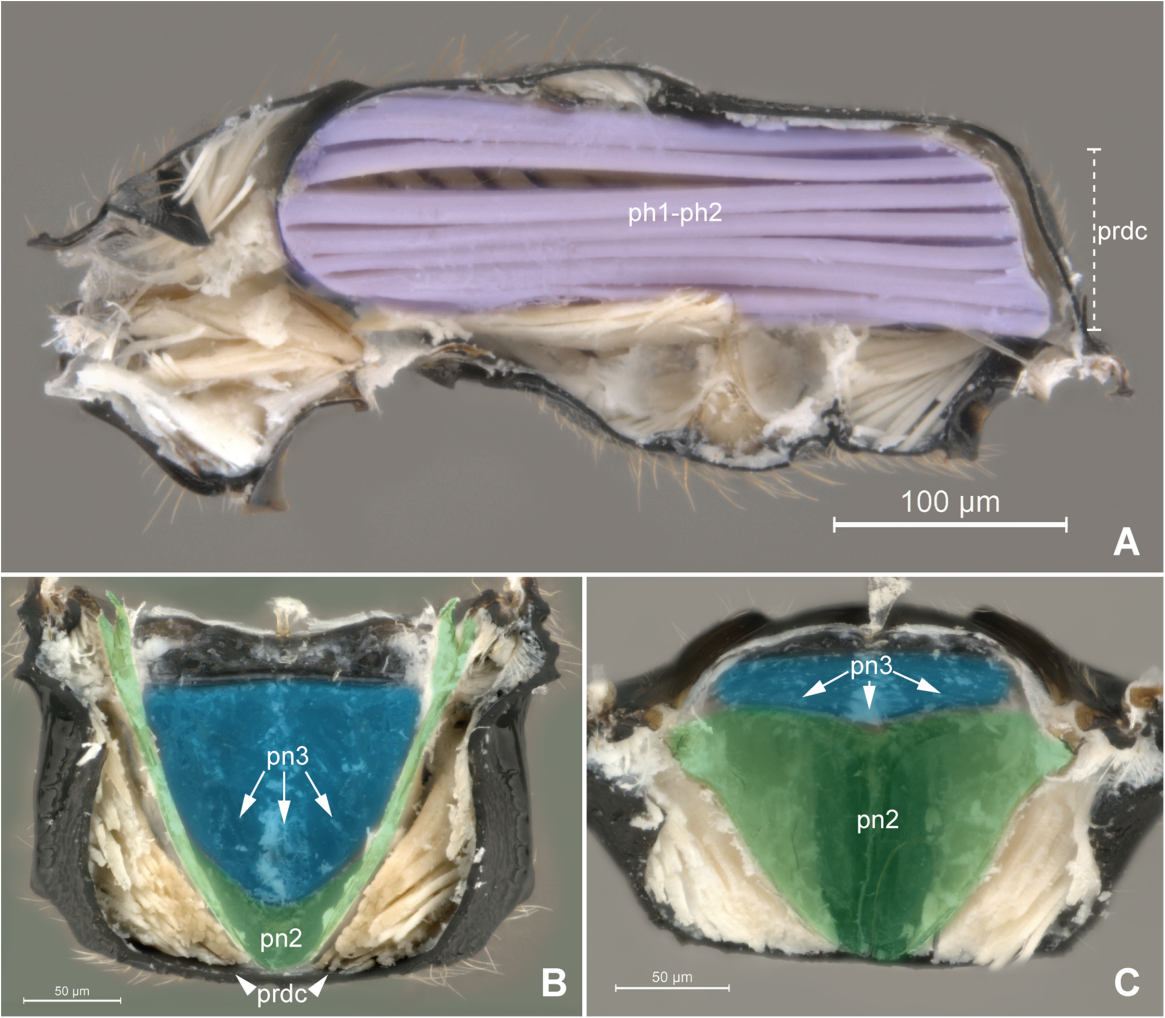
Detail of Meso- and metapostnotum. 6A *Pseudisobrachium* sp. (Pristocerinae), 6B-C *Chlorepyris* sp. (Epyrinae). (A) Mesosoma, internal medial view. Purple = prophragmo-mesophragmal muscle. (B) Metanotum and metapectal-propodeal complex, internal ventral view. Blue = metapostnotum; green = mesopostnotum. (C) Metapectal-propodeal complex, internal anterior view. Blue = metapostnotum; green = mesopostnotum.

In general, the external limits of the metapostnotum can be observed by the internal shape of the mesopostnotum (**pn2**; Fig 3B). In Bethylidae, the mesopostnotum can be described as having a “U” shape and is compounded by the **mesolaterophragma** (**lph2**; Figs 4 and 5) and **mesophragma** (**ph2**; Figs 4 and 5). Also in Bethylidae, the mesopostnotum connects with the **mesopleuron** (**pl2**) through the mesolaterophragma, and extends through a long and narrow ‘handle’, which forms a gap between the **mesoscutellum** (**scl2**; Figs 4 and 5) and propodeal declivity. This ‘handle’ undergoes a twist and expands posteriorly to form a membranous area (mesophragma), which is arranged perpendicularly [5] to the longitudinal axis of the body, and attaches the prophragmo-mesophragmal muscle (**ph1-ph2**; Fig 6A). The prophragmo-mesophragmal muscle covers the highest percentage of medial area of the metapectal-propodeal complex and occupies the entire area below the metapostnotum, leaving only the surrounding areas for the other muscles associated with locomotion and metasomatic movement.

The external limits of the metapostnotum can be differentiated from the propodeum by the sculpturing (Pristocerinae), flatness (Bethylinae) and (or) pigmentation of the integument (Scleroderminae). Additional structures may contribute to delimitating the metapostnotum, such as the **transverse anterior carina of the propodeum** (**tac**; Fig 2C and 2D), which extends along the anterior margin and fuses with the **metapleural arm** (**pl3ar**; Fig 2C) anterolaterally anterior of the propodeal spiracle; metapostnotal-propodeal suture, which is often composed of a carina; and transverse posterior carina of the propodeum anterior to the propodeal declivity (Fig 6A and 6B). Certain bethylids groups posses a **metapostnotal median carina** (**pn3mc**; Figs 1A–1C, 2C and 2D), which divides the metapostnotum into two larger areas, or an anteromedial elevation, but they do not correspond to an internal muscle insertion point.

In all bethylid subfamilies, and Hymenoptera in general, the **metaphragma** (**ph3**; Fig 2B) originates from the metapostnotum, being represented only by its lateral remnants, as a narrow strip in the anterolateral margin in the metapleural arm [5], and can only be identified after dissection of the metapectal-propodeal complex. However, according to our observations, internally, the medial portion of the metaphragma is completely modified to accommodate the metaphragmo-second abdominal tergal muscle posteriorly, since the metapostnotum has been expanded to the propodeal declivity (Fig 6A–6C).

**Fig 7:**
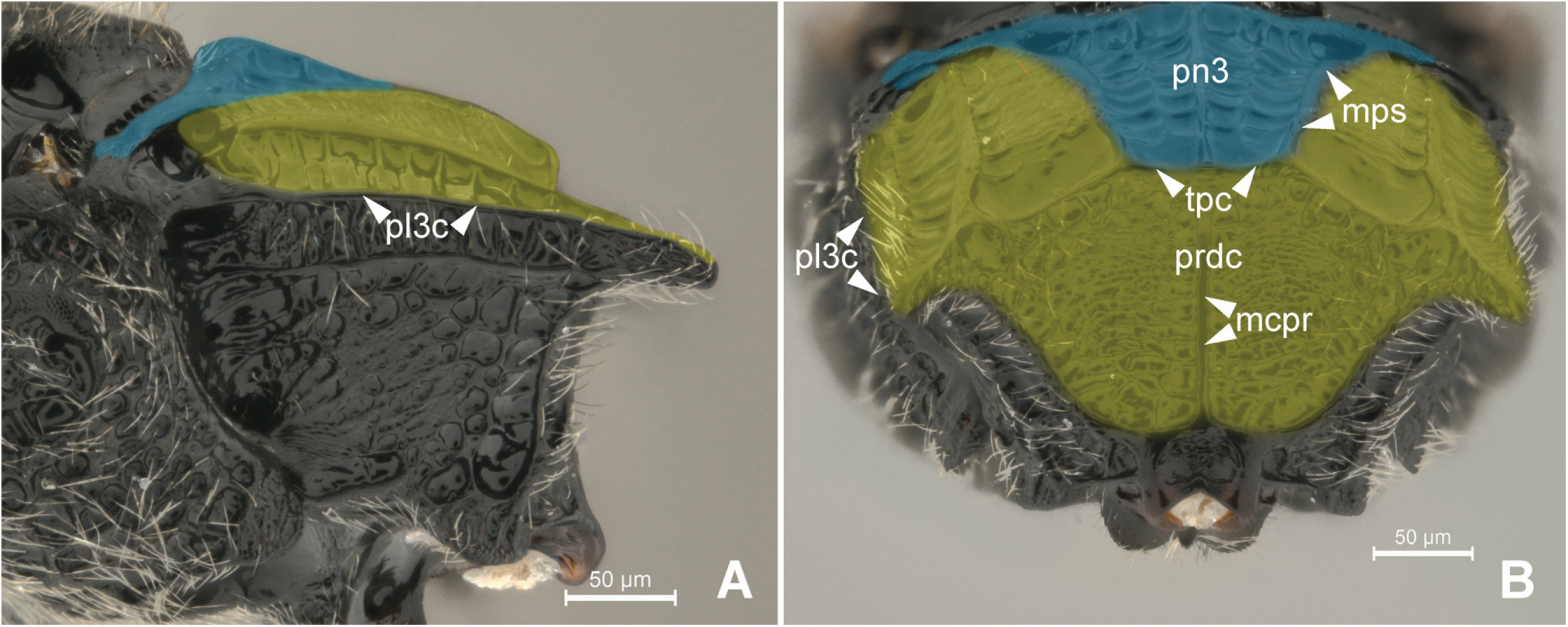
Detail of Metapectal-propodeal complex. 7A-B *Heterocoelia* sp. (Mesitiinae). (A) Metapectal-propodeal complex, lateral view. Blue = metapostnotum; yellow = first abdominal tergo. (B) Metapectal-propodeal complex, posterior view. Blue = metapostnotum; yellow = first abdominal tergo.

The first abdominal tergum has been medially invaded by the metapostnotum (Fig 6B and 6C), and it is now dorsally restricted to a region that extends between the propodeal spiracle, external lateral area of the metapostnotal-propodeal suture, and the entire propodeal declivity (Fig 1A–1C). The dorsal area of the propodeum (metapostnotum + T1) is separated from the lateral area (metapleura+T1) by the **metapleural carina** (**pl3c**; Figs 2C, 2D, 7A and 7B). Laterally, the propodeum is mainly formed by the metapleuron; however, there is no clear delimitation between its two components (metapleuron and T1).

In general, the metapostnotal-propodeal suture defines the metapostnotum in Bethylidae, and this suture can be either well defined (Fig 1A and 1B) or obliterated (Fig 1C and 1D) depending on the sculpturing, suture length variation (usually absent in the posterior third), and integument flatness.

In Bethylidae (Fig 2A), the metapostnotum varies and can be an important structure for recognizing lineages within each subfamily. In Bethylinae, the metapostnotum posses different shapes; for example, an elevated medial region in *Bethylus* Latreille; pronounced sculpture or unevenness in *Eupsenella* Westwood; elevated anteromedial region or fine unevenness in *Goniozus* Föster; pronounced suture and/or unevenness in *Odontepyris* Kieffer; elevated anteromedial region, pronounced suture and unevenness in *Prosierola* Kieffer; and tenuous sculpture or unevenness in *Sierola* Cameron. Polaszek and Krombein [21] described the anteromedial elevation in *Goniozus* and *Prosierola* as a different structure than the “propodeal triangle" found in Apidae and Sphecidae [5]. In fact, the anteromedial elevation cannot be compared to the “propodeal triangle” found in Apoidea because the metaphragmo-second abdominal tergal muscle of *Goniozus* and *Prosierola* follows the general pattern of Bethylidae.

Each bethylids subfamily has a general pattern for the metapostnotum, but with specific characteristics for each group. In all genera of the Epyrinae (Fig 1B) the metapostnotum is differentiated from the propodeum by the pronounced metapostnotal-propodeal suture, and distinct sculpturing. In all genera of the Mesitiinae (Fig 1A), the metapostnotum is differentiated from the propodeum by pronounced sculpturing and unevenness of the area. In almost all Pristocerinae (Fig 1C) genera, a rugulose metapostnotum area with different sculpturing levels is often present, and a triangle is formed (referred to by Evans [22] as the basal triangle) with texture distinct from that of the propodeum. In Scleroderminae (Fig 1D), the metapostnotum is completely indistinguishable from the propodeum, and *Pararhabdepyris* Gorbatovskii have differences of sculpturing, which is also observed in all the representatives of Pristocerinae. In certain cases, the boundaries between the metapostnotum and first abdominal tergum are not clear; therefore, observations of the characteristics, such as the presence of carinas, differences in sculpturing and unevenness in integument, may assist in performing accurate delimitations.

## Discussion

### Metapostnotum in Bethylidae

The expansion of the metapostnotum in Bethylidae (Fig 7B) allowed for the elongation of the mesopostnotum (Fig 6B and 6C) and mesophragmo-metaphragmal muscle (**ph1-ph2**). Mesophragmo-metaphragmal muscle action, associated with the first mesopleuron-mesonotal (**pl2-t2a**), changes the volume of mesosoma, and in combination with direct-flight muscle action, these changes lead to the movement of the forewings. Therefore, the elongation of ph1-ph2 most likely positively influences the flight of bethylids because flight resistance tends to show a strong positive correlation with relative muscle size [23].

Most of studies that indicate a reduction and/or absence of the metapostnotum (e.g., [4, 5, 24]) only used the ph2-ph3 muscle as a reference (Figs 2B and 3A). According to Snodgrass [1], insects that have reduced metapostnotum due to reduced mesophragmo-metaphragmal muscle (**ph2-ph3**; Figs 2B and 3A), resulting in more developed forewings than hind wings. However, the metaphragmo-second abdominal tergal muscle (**ph3-T2**; Fig 3A) also attaches to the metapostnotum and is not related to flight but to metasomal movement, which results in, the elongation of the metapostnotum in insects with reduced posterior wings. The ph3-T2 is a convergent muscle that has a broader base relative to the distal insertion, giving the muscle a triangular shape, providing greater versatility [25], since stimulation of only one part of the muscle produces movement in different directions.

The metapectal-propodeal complex in Bethylidae supports wing flapping by the prophragmo-mesophragmal, and first mesopleuron-mesonotal muscles as well as for metasomal movement by the metaphragmo-second abdominal tergal muscle, and propodeo-second abdominal segment muscle (**T1-S/T2**; Figs 3A and 2B).

Pristocerinae flat wasps have extreme sexual dimorphism with winged males and wingless females [26]. This condition allows copulation during the nuptial flight, which is referred to as phoretic copulation [27]. During the phoretic copulation event, the male must carry the female through the attachment of both abdomens in flight. The metapectal-propodeal complex must maintain overall insect stability to enable phoretic copulation, so that several tasks can be performed, including those directly related to balance (which require posterior leg movement and abdominal support while carrying the female), and indirectly related to balance (structural reinforcement of the mesophragma during mesothoracic deformation of wing flapping).

According to our observations, wingless females of Pristocerinae usually exhibit complete fusion of the metathoracic segment with the first abdominal segment (*Acrepyris* Kieffer, *Apenesia* Westwood, *Dissomphalus* Ashmead, *Pseudisobrachium* Kieffer, *Pristocera* Klug, *Prosapenesia* Kieffer and *Scaphepyris* Kieffer), and some Scleroderminae (*Cephalonomia* Westwood); in some *Sclerodermus* Latreille, there is a separation between the metanotum and propodeum (metathorax + first abdominal segment). Evidence of flight musculature, such as the presence of mesophragma and associated structures, is not observed in apterous females of Pristocerinae and some genera of Scleroderminae (unpublished data). Similar to the males of the Scleroderminae, all the females are devoid of metapostnotum differentiation in the propodeal disc.

### The evolution of the metapostnotum in Bethylidae in light of the available phylogenetic hypothesis

Terayama [28] is the only author to indicate that Bethylinae are the second-most basal subfamily in Bethylidae. The most recent analyses of Carr et al. [29] and Alencar and Azevedo [12] corroborate the previous work of Evans [22], Sorg [30] and Carpenter [31], who found that Bethylinae are the most basal subfamily in Bethylidae. The representatives of Bethylinae exhibit all the metapostnotum variations found in the family, as illustrated by *Prosierola*, which have two foveae in the anterior region and lateral unevenness of the metapostnotum (Fig 1A).

Pristocerinae are phylogenetically unstable and can be observed in different phylogenetic positions in the family: Terayama [28] identified as the most basal group, Carr et al. [29] as the second-most basal and sister group of all Epyrinae (Epyrini), and Alencar and Azevedo [12] as the sister group of Mesitiinae. A few genera within the subfamily show a subsequent modification of the ground plan condition, with the expansion of sculpturing toward the propodeum in *Pristocera*, and its complete obliteration in *Neoapenesia* Terayama.

Mesitiinae showed increased phylogeny stability relative to Pristocerinae and were identified as a sister group of Epyrinae (Epyrini + Sclerodermini) in Evans [22], Sorg [30], Carpenter [31] and Terayama [28] and a sister group of Sclerodermini (Scleroderminae) [29], and Pristocerinae [12]. Sclerodermini were revalidated as a subfamily by Alencar and Azevedo [12], and may have a close relationship with Mesitiinae as a sister group [12, 29]. According to the data obtained in the current study, the reduction of metapostnotum markings in the metapectal-propodeal complex may be considered the ground plan for this subfamily, which includes possible reversals, as found in *Pararhabdepyris*. Alencar and Azevedo [12] previously noted the absence of metapostnotum differentiation in the diagnosis of the subfamily interpreted as “propodeal disc with or without carinae."

Sorg [30] and Carpenter [31] discuss some evidences of paraphyly of Epyrinae (Epyrini + Sclerodermini), evidently in terms of Mesitiinae. Alencar and Azevedo [12] suggested that the propodeum with a transverse anterior carina, carinate disc (= metapostnotal-propodeal suture represented by carinae), median carina almost always complete (= metapostnotal median carina), and posterolateral angle with a fovea, are part of the diagnosis of the subfamily. Epyrinae are the largest subfamily of Bethylidae and the most confusing in terms of the relationships between genera, within the Epyrinae core, referred to as “clade A” by Alencar and Azevedo ([12]: see Fig 100). Inside the core, *Epyris* Westwood is the most speciose genus and have a wide range of metapostnotum types that most likely represent more than one internal lineage of the taxon. In the same study, three clades considered monophyletic were retrieved in all analyses, and the metapostnotal-propodeal suture was distributed as follows: (*Laelius* + *Anisepyris*) have a posteriorly interrupted metapostnotal-propodeal suture; (*Holepyris* + (*Formosiepyris* + *Disepyris*)) and (*Formosiepyris* + *Disepyris*) exhibit a posteriorly complete suture; and *Holepyris* Kieffer have a nearly posteriorly interrupted suture, which is an intermediate condition to what was found in the two more inclusive clades (certain species from Madagascar are similar to the *Laelius* Ashmead pattern).

The metapostnotum together with other characteristics can provide further evidence of potentially erroneously positioned taxa in other subfamilies, such as *Glenosema* Kieffer, which belongs to the Scleroderminae but has a similar configuration to that found in Epyrinae; this hypothesis was suggested by Lanes and Azevedo ([28]: Figs 5, 6 and 7). In Bethylinae, Alencar and Azevedo [32] reported *Odontepyris acrius* as the only species with an anteromedial elevation of the propodeum. In our interpretation this feature indicates that this species is, in fact, more related to *Goniozus* (unpublished data).

### Implications for homology assessments in Chrysidoidea

**Fig 8:**
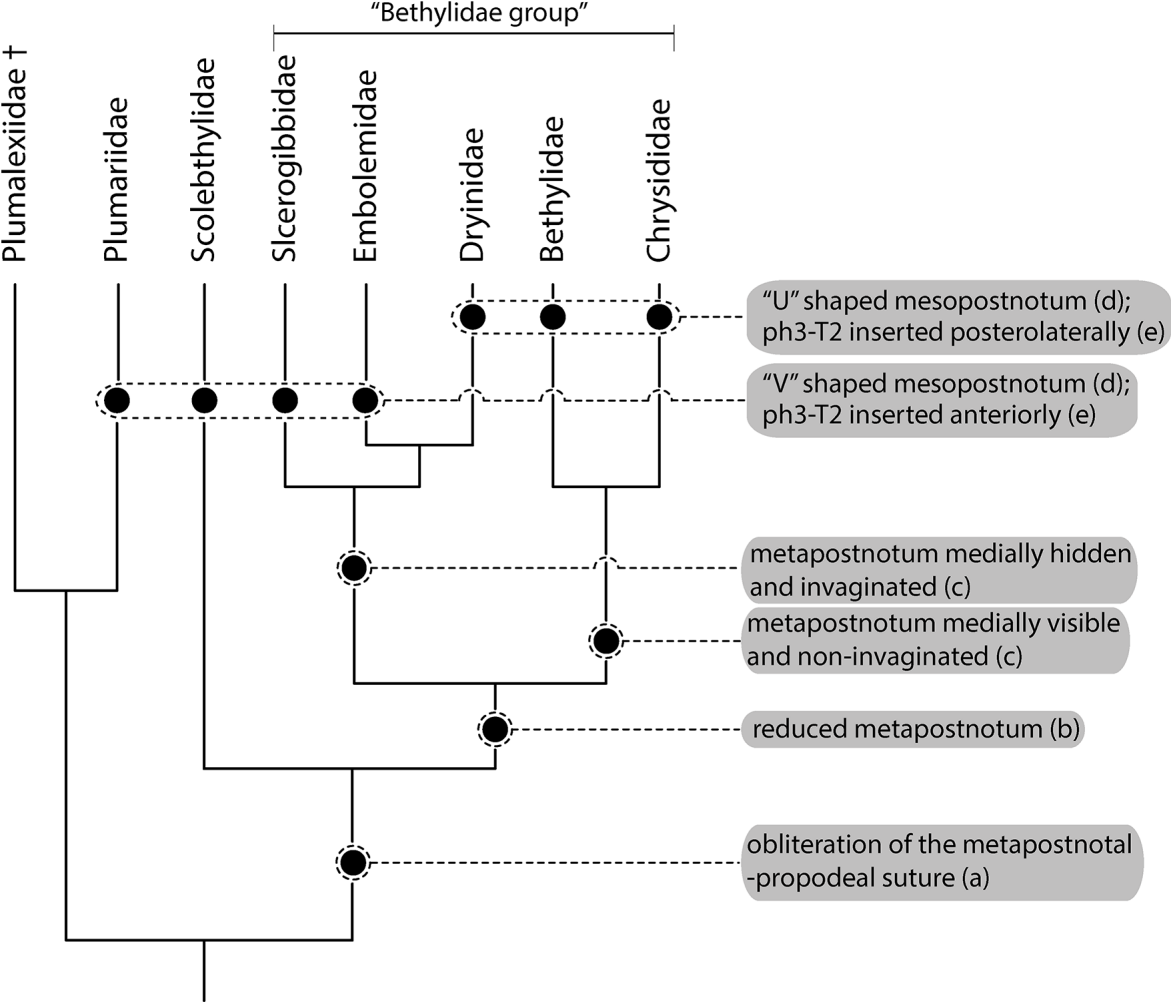
Cladogram of families of Chrysidoidea modified from Brothers ([36], Fig 22). Black hatchmarks indicate shared state according: (a) Brothers [37]; (b) Brothers [37], Carpenter [31, 33], and Brothers and Carpenter [34]; (c) Brothers and Carpenter [34]; (d) present work; (e) Whitfield et al [4].

Based on the current knowledge of the relationship between the families of Chrysidoidea, several authors have attempted to understand their relationships [31, 33, 34, 35, 36]. The literature suggests that Plumariidae are sister-group to the remaining families, which form a monophyletic group, and suggest that Scolebythidae are a sister group of the “Bethylidae group” ((Sclerogibbidae + (Embolemidae + Dryinidae)) + (Bethylidae + Chrysididae)) (Fig 8). Brothers [37], Carpenter [31, 33] and Brothers and Carpenter [34] observed that a reduced metapostnotum was a synapomorphic state in (Bethylidae + Chrysididae) + (Sclerogibbidae + (Embolemidae + Dryinidae)). According to Brothers and Carpenter [34], Bethylidae + Chrysididae present a medially visible and non-invaginated metapostnotum, whereas in Sclerogibbidae + (Embolemidae + Dryinidae), the metapostnotum is medially hidden and invaginated (Fig 8).

Brothers [37] noted that the synapomorphies of Scolebythidae and the “Bethylidae group” include a metapostnotum reduced to its lateral remnant and obliteration of the metapostnotal-propodeal suture. Rasnitsyn [38] suggested that basal Dryinidae (Aphelopinae and Anteoninae) have a metapostnotal-propodeal suture; however, the results of Carpenter [33] and Olmi [39] indicate that the suture was not observed in any of the internal members of the family. Carpenter [33] considered Rasnitsyn’s results to be mistaken and suggested that the suture obliteration and resulting metapostnotum constriction were a synapomorphy for Bethylidae, Chrysididae, Dryinidae, Embolemidae, and Sclerogibbidae, as well as a convergent state in certain Scolebythidae. According to our observations in the current study, Rasnitsyn's findings may be correct if the internal rearrangement of the musculature and structures associated with the mesopostnotum and metapostnotum are considered; these characters were not investigated by Carpenter [33] and Olmi [39].

Scolebythidae and Sclerogibbidae have a ground plan similar to that of Plumariidae, with a mesopostnotum similar to that of Apocrita with a “V” shape, antecostal sulcus in the first abdominal segment, and ph3-T2 muscles originating from the anterior margin of the propodeum similar to that of non-aculeate Apocrita [4]. Furthermore, Embolemidae, Dryinidae and Chrysididae have external and internal characteristics similar to that of Bethylidae, including a “U”-shaped mesopostnotum and ph3-T2 muscle inserted posterolaterally in the propodeum; these characteristics suggest that these families share the same morphology, and Sclerogibbidae derived a convergent state internal to the clade currently accepted as (Sclerogibbidae + (Embolemidae + Dryinidae)) + (Bethylidae + Chrysididae) (Fig 8). All the main characters elucidated above were summarized in modified cladogram of Brothers [36] with shared state according to several authors [4, 31, 33, 34, 37]. A more detailed study of the muscles associated with the metapostnotum can resolve the confusion within Chrysidoidea.

### The mesophragmo-metaphragmal muscle suggests a new hypothesis for metapostnotum evolution in Apoidea and Bethylidae

**Fig 9:**
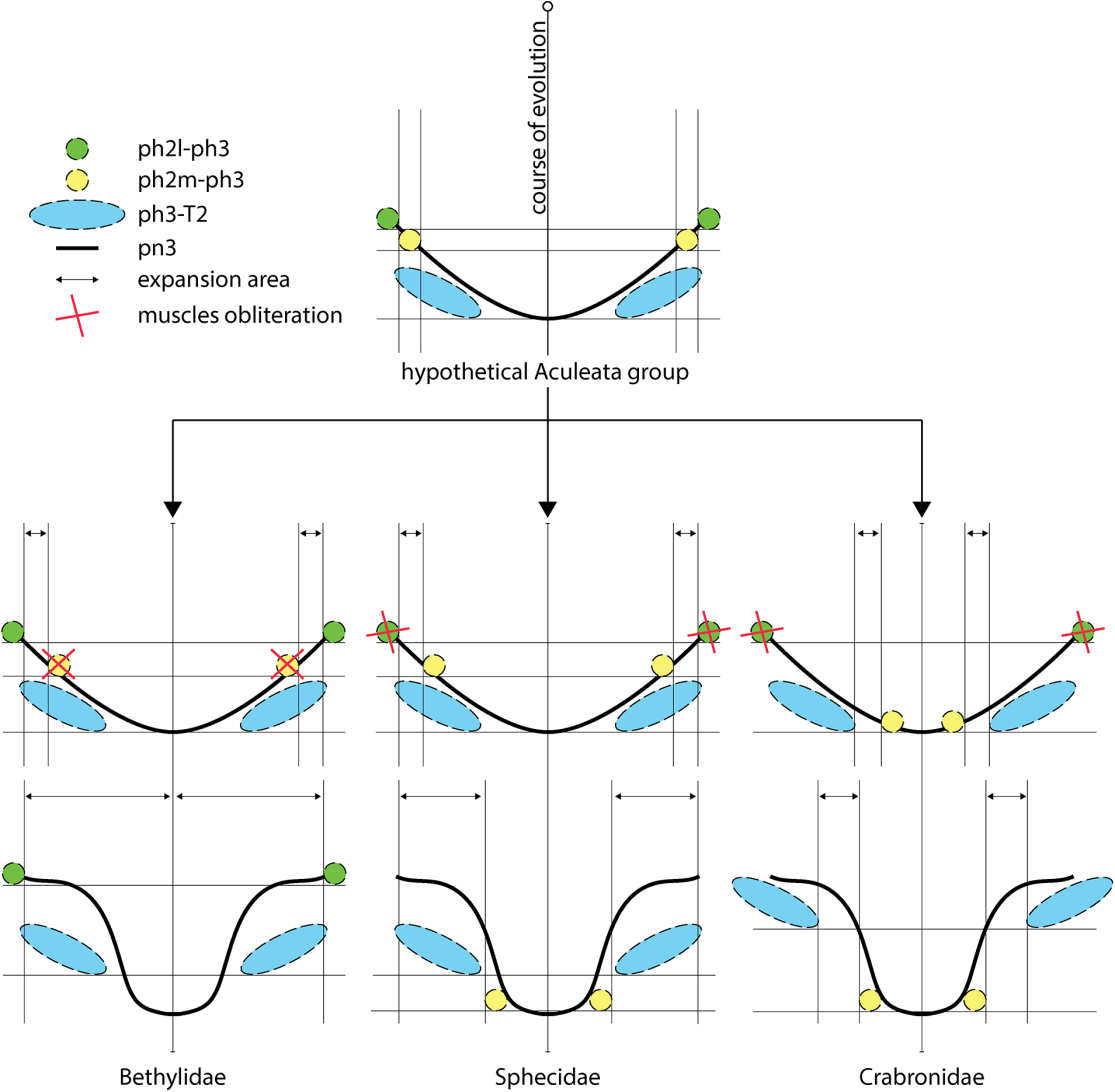
Evolutionary hypothesis about displacement of ph2-ph3 muscles. Green circle = lateral mesophragmo-metaphragmal muscle (ph2l-ph3); yellow circle = median mesophragmo-metaphragmal muscle (ph2m-ph3); blue ellipse = metaphragmo-second abdominal tergal muscle (ph3-T2); solid line = metapostnotum; double arrow = expansion area; red X = muscles obliteration.

Vilhelmsen [6] considered the mesophragmo-metaphragmal muscle subdivision as an apparent autapomorphy for Tenthredinoidea. Subsequently, Vilhelmsen et al. [7] renamed this muscle as median (ph2m-ph3) and lateral (ph2l-ph3) mesophragmo-metaphragmal muscle, and they considered the ph2l-ph3 is exclusive to Tenthredinoidea. The medial and lateral subdivision of the mesophragmo-metaphragmal muscle suggests a new hypothesis for metapostnotum evolution in Apoidea and Bethylidae, which is similar in terms of the expansion observed in both groups, but with different processes. According to the position of the mesophragmo-metaphragmal muscle in Bethylidae (similar to the position it occupies in other Apocrita) the evolution of this muscle occurred through the obliteration of the median muscle (ph2m-ph3) and permanence of the mesophragmo-metaphragmal lateral muscle only (ph2l-ph3l), which allowed for the expansion of the area of metapostnotum where the two muscle bundles of the ph2m-ph3 muscle would be located, and posterior displacement of the ph3-T2 muscle (Fig 9). In turn, the median muscle (ph2m-ph3) in Apoidea was retained, the lateral muscle (ph2l-ph3) was obliterated, and metapostnotum expanded in two distinct regions: an anterior region (Sphecidae; Fig 9) between the two muscle bundles of the ph2-ph3 muscle, which is similar to Bethylidae but promotes the displacement of the ph2m-ph3 and ph3-T2 muscles to their current condition, and a posterior region (Crabronidae; Fig 9) between the ph3-T2 and ph2m-ph3 muscles, with the ph3-T2 muscle maintaining its position and ph2m-ph3 muscle displaced posteriorly. However, if we consider that the subdivision of the ph2-ph3 is an autapomorphy of Tenthredinoidea, then the ph2-ph3 muscle does not need to be differentiated as median or lateral in the other Apocrita, as both sections would be included in the ph2-ph3 muscle from which the ph2m-ph3 and ph2l-ph3 originated. Because the ph2-ph3 muscle in bethylids follows the same pattern as most Apocrita, a more detailed investigation of the identity of the ph2-ph3 muscle in Apoidea could clarify its evolution.

## Conclusion

The present analyses represent the most comprehensive effort in bethylid wasps based on a propodeum anatomical structures (sclerites and muscles) data set to date. We have a new interpretation to the anteromedian area of the propodeal disc sensu Evans [22] as the metapostnotum. We have demonstrated that the metapostnotum (**pn3**) in Bethylidae was medially expanded in the propodeal disc, resulting a variety of morphologically distinct forms, and each subfamily has a general pattern. We have demonstrated the currently relationship of families within Chrysidoidea to be well corroborated. In particular, Embolemidae, Dryinidae and Chrysididae share most of morphological similarities observed in meso- and metapostnotum of Bethylidae. Based on a subdivision

Originally, morphological studies on insects were general and complex and consisted of large manuscripts that attempted to clarify the entire structural pattern of the class (e.g., [1, 29]). However, several researchers have begun to restrict their studies to specific taxa, and they have performed more detailed studies with lower taxonomic coverage and a lower perception of homologies and patterns. Alternatively, other researchers have restricted their studies to specific characters and investigated large taxonomic groups through the analysis of only one character or a set of associated characters (e.g., [40]). The ideal scenario would include investigations that were structurally and taxonomically complete, studies similar to those originally proposed by Snodgrass; however, such studies would require a great deal of time and a significant number of experts. Thus, studies that focus on a single structure contained in a larger number of taxa are more suitable and present specific questions that can contribute to broader issues, thus providing a better understanding of the morphology and evolution of insects. As previously proposed by Gibson [10], substantial progress in Hymenoptera phylogeny will not only require new and better computer algorithms but also a renaissance in comparative morphology to analyze alleged transformation series.

## Supporting Information

S1 Table Specimens dissected and deposition information for specimen data. (XLSX)

S2 Table List of ontological concepts of anatomical structures applied in the present work, and a brief historical review of the terms. The table was generated by the analyze tool available through the Hymenoptera Glossary (HAO), using the Universal Resource Identifiers (URIs). New terms highlighted with an asterisk (*), terms not used in the Hymenoptera glossary (**), and synonymous ones marked with an equal sign (=).
(XLSX)
